# Optimization of PTB7-FTh-Based Organic Photovoltaic Devices Using Blade Coating: Influence of Solvent, Acceptor Composition, and Annealing on Performance

**DOI:** 10.1021/acsomega.5c07832

**Published:** 2025-09-10

**Authors:** Isabela Custódio Mota, Renata da SilvaCardoso, Igor Tenório Soares, Lucas Galhardo Pimenta Tienne, Tamires Y. G. Alves, Letícia A. Marcate, Juliana L. S. Martins, Gabriela A. Soares, Bárbara H. S. Miranda, Diego Bagnis, Erica G. Chaves, Renata P. Azevedo, Danielle C. P. Freitas, Marlito G. Junior, Maria de Fátima Vieira Marques

**Affiliations:** † Instituto de Macromoléculas Professora Eloisa Mano (IMA), 28125 Universidade Federal do Rio de Janeiro, Av. Horácio Macedo, 2030, CT-Bloco J, Ilha do Fundão, Rio de Janeiro, Rio de Janeiro CEP 21941-598, Brazil; ‡ ONINN Innovation Center (Centro de Inovações), Avenida Jose Candido da Silveira, 2000, Horto Florestal, CEP 31035536 Belo Horizonte, Minas Gerais, Brazil; § Leopoldo Americo Miguêz de Mello Research and Development Center, 42506 Federal University of Rio de Janeiro (PETROBRAS/CENPES), Av. Horácio Macedo, 950, Ilha do Fundão, Rio de Janeiro, Rio de Janeiro CEP 21941-915, Brazil

## Abstract

In this study, we report the synthesis and characterization of a fluorinated polymer, PTB7-FTh, designed as a donor material for organic photovoltaic (OPV) devices. The polymer structure is based on PTB7-Th, with the addition of fluorine substituents to enhance molecular interactions and energy level alignment. OPV devices were fabricated using a scalable blade coating technique under an ambient atmosphere, utilizing different solvent systems, donor-to-acceptor ratios, and acceptor materials (Y6 and PC_71_BM). The effects of active layer thickness and thermal annealing on device performance were also investigated. The optimized ternary blend system, incorporating PTB7-FTh, Y6, and PC_71_BM (1:1.6:0.2), exhibited a power conversion efficiency (PCE) of 5.03%, with an open-circuit voltage (*V*_oc_) of 0.76 V, a short-circuit current density (*J*_sc_) of 13.39 mA cm^–2^, and a fill factor (FF) of 49.22%. The system PTB7-FTh:Y6:PCBM, 12 mg mL^–1^ in chloroform, 1:1.6:0.2, pretreatment heating at 80 °C, named CF system, demonstrated superior charge transport and light absorption properties, particularly after thermal annealing, which led to efficiency improvements. The use of o-xylene as a processing solvent proved advantageous for scalability while maintaining competitive device performance compared to those fabricated under inert conditions. This study highlights the potential of PTB7-FTh as a promising donor material and underscores the importance of solvent selection, acceptor composition, and processing conditions for advancing the scalability of OPV technology.

## Graphical abstract

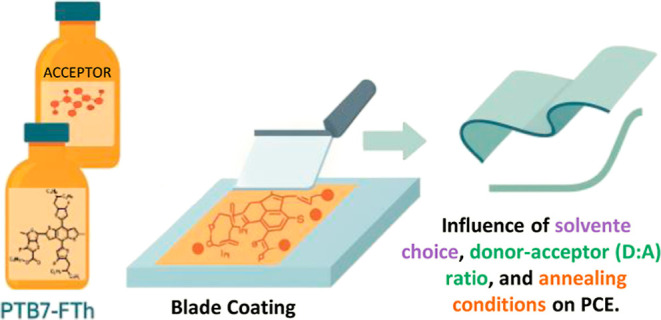

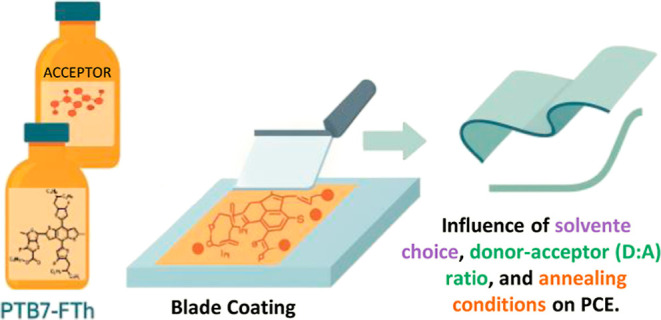

## 1 Introduction

Polymeric photovoltaic energy emerges as a promising solution to energy scarcity.^1^ Unlike conventional solar cells, which use silicon, polymeric cells are simpler to produce, flexible, and scalable, making their implementation easier in various environments, including remote areas.^2^ In addition to being a renewable and environmentally cleaner alternative, this technology has the potential to democratize access to energy, making photovoltaic generation more accessible and sustainable.^3^

Photovoltaic devices typically feature an active layer with a p-type semiconductor polymer as the donor material and an n-type organic semiconductor as the acceptor.^4^ For a long time, fullerene derivatives, such as PCBM, were the primary acceptor materials due to their good electron acceptance.^5^ However, due to limitations in stability and efficiency, nonfullerene acceptors (NFAs) have emerged as a promising alternative, offering greater flexibility in modulating optical and electronic properties, which can improve device efficiency and performance.^6^

It is well-known that energy levels, absorption coefficients, and morphology can be subtly manipulated by modifying the chemical structures of donor (D) and acceptor (A) units, both in acceptor materials—making NFAs so promising—and in donor materials.^7^ Kan and collaborators developed a new acceptor molecule of the donor–acceptor–donor (A–D–A) type, called NCBDT, by modifying the D and A units of NFBDT. The addition of an octyl group to the D unit raised the HOMO energy level, while fluorination of the A units lowered the LUMO energy level. The resulting acceptor shows an optical bandgap of 1.45 eV, extending the absorption range to approximately 860 nm, in the near-infrared region.^8^ In polymer solar cells (PSCs), manipulating the Frontier orbital energy levels of photovoltaic materials through chemical modifications is critical for increasing the open-circuit voltage (*V*_oc_),^9^ as their values are approximately directly proportional to the gaps between the HOMO levels of electron donors and the LUMO levels of electron acceptors in their active layers. Therefore, *V*_oc_ can be effectively enhanced by raising the LUMO levels of acceptors and/or lowering the HOMO levels of donors.^10^

Chang et al., 2018 developed a narrow-bandgap copolymer, PBFTT, incorporating 4,8-bis­(5-(2-ethylhexyl)-4-fluorothiophene-2-yl)­benzo­[1,2-*b*:4,5-*b*′]­dithiophene (BDT-2F) as the donor unit and octyl-3-fluorothieno­[3,4-*b*]­thiophene-2-carboxylate (TT) as the acceptor unit. This copolymer was used as a donor material in nonfullerene organic photovoltaic (NFA-OPV) devices. When comparing PBFTT with its analogous polymer, PTB7-Th, the authors observed that PBFTT exhibited a similar absorption spectrum with a slight blue shift, a deeper HOMO energy level of −5.47 eV, and a slightly higher hole mobility. OPV devices were fabricated in a glovebox-controlled atmosphere using PBFTT:ITIC as the active layer, deposited by spin-coating, with an active of 0.4 cm^2^. The PBFTT:ITIC-based OPV device achieved a power conversion efficiency (PCE) of 9.1%, with an open-circuit voltage (*V*_oc_) of 0.94 V, a short-circuit current density (*J*_sc_) of 16.0 mA cm^–2^, and a fill factor (FF) of 60.5%. In contrast, the PTB7-Th:ITIC-based device showed a lower PCE of 6.8%, with a *V*_oc_ of 0.81 V, a *J*_sc_ of 14.2 mA cm^–2^, and an FF of 59.1%. These results highlight the positive influence of fluorine incorporation into the polymer structure. The introduction of fluorine atoms enhanced intermolecular interactions through increased dipole moments, leading to improved molecular packing and charge transport.^12^ Additionally, fluorination contributed to a deeper HOMO energy level, which increased the *V*_oc_ and overall efficiency of the device.^13^ The improved charge mobility observed in PBFTT further underscores the role of fluorine in optimizing the polymer backbone for efficient charge transport. Consequently, the study demonstrates that strategic fluorination can be an effective approach for designing high-performance donor materials in NFA-OPV devices.

To investigate the synergistic effects of alkylthio-substitution and fluorination on molecular orbital levels, Du and collaborators introduced two new D–A polymers based on benzo­[1,2-*b*:4,5-*b*′]­dithiophene (BDT) as the donor unit, simultaneously alkylthiolated and fluorinated: PBDTT–SF–TT and PBDTT–SF–BDD. These polymers combine alkylthiolation and fluorination in the BDT branches, using 2-ethylhexyl-3-fluorothieno­[3,4-*b*]­thiophene-2-carboxylate (TT) or 1,3-bis­(thiophen-2-yl)-5,7-bis­(2-ethylhexyl)­benzo­[1,2-*c*:4,5-*c*′]­dithiophene-4,8-dione (BDD) as electron-accepting units. The solar cells made from these materials show high open-circuit voltages (*V*_oc_) of 1.00 V (PBDTT–SF–TT) and 0.97 V (PBDTT–SF–BDD), associated with their deep HOMO levels of −5.54 and −5.61 eV, respectively.^14^

In the present work, a similar copolymer was synthesized, resembling PBFTT; however, the branching in the acceptor unit of the polymer differs (structures in Figure 1). This copolymer was designated as PTB7-FTh. OPV devices with an area of 0.55 cm^2^ were manufactured using the blade coating technique in an uncontrolled laboratory atmosphere, employing various configurations to enhance efficiency. Additionally, the fabrication process was designed to replicate conditions closer to those used in industrial-scale photovoltaic device production, ensuring scalability.

**Figure 1:**
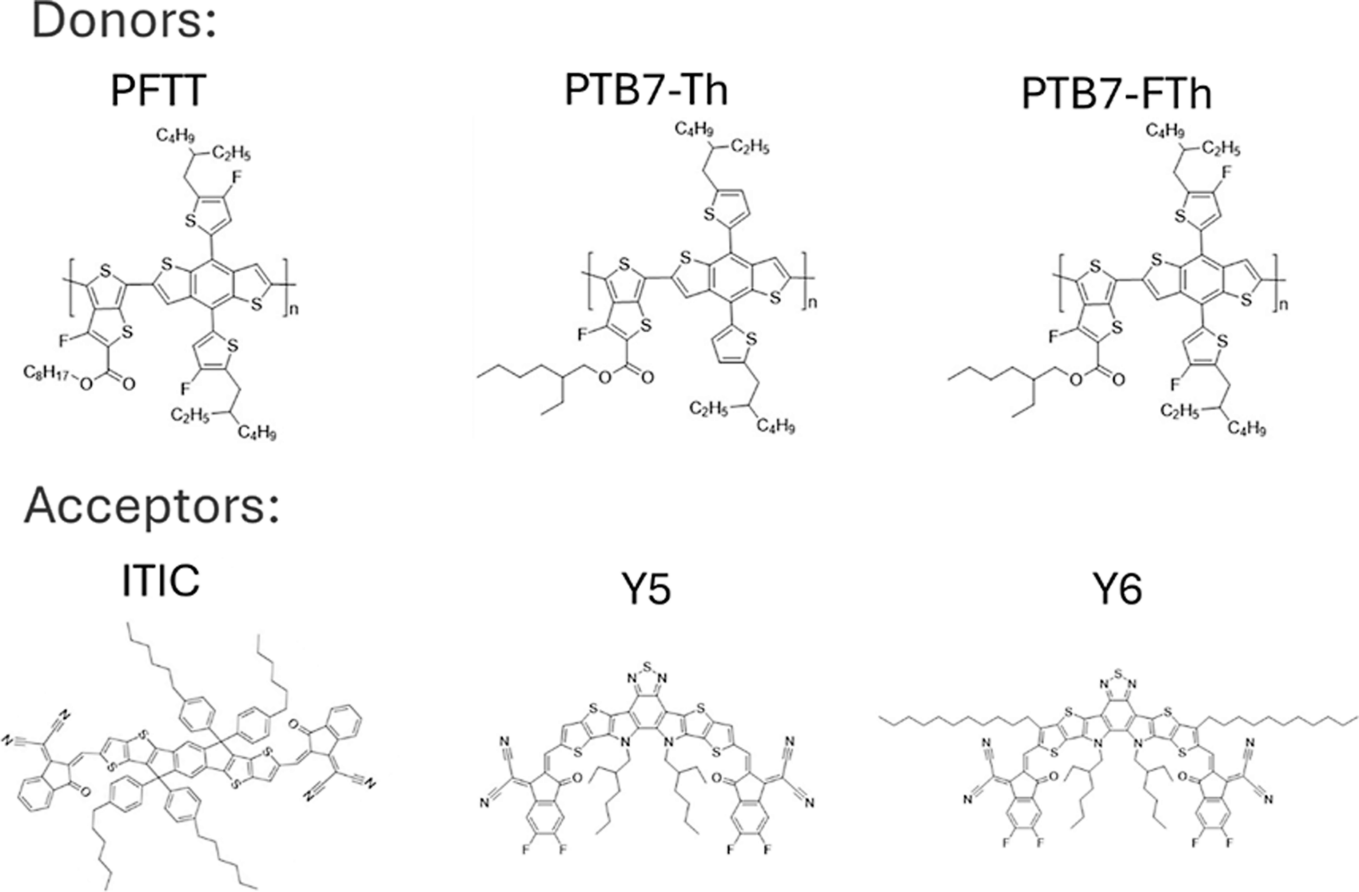
Structures of donor and acceptor materials for the active layer of OPV solar cells.

## 2 Experimental Section

### 2.1 Chemicals

All solvents were of reagent grade and purchased from Alfa Aesar Synth Shop (USA). Reagents were obtained from Alfa Chemical Co., Ltd (China). Zinc oxide (ZnO) was supplied by InfinityPV ApS (Denmark), and the modified PEDOT, marketed as HTL-X, was provided by Raynergy Tek Inc. (Taiwan).

### 2.2 Synthesis of the Polymer PTB7-FTh

In a 25 mL round-bottom flask, 4,6-dibromo-3-fluorothieno­[3,4-*b*]­thiophene-2-carboxylic acid 2-ethylhexyl ester (0.2 mmol) (M1) and 2,6-bis­(trimethytin)-4,8-bis­(5-(2-ethylhexyl)-4-fluorothiophen-2-yl)­benzo­[1,2-*b*:4,5-*b*′]­dithiophene (0.2 mmol) (M2) were dissolved in 5 mL of distilled toluene and 1 mL of DMF bubbled with nitrogen for 10 min. Ten mg of Pd­(PPh_3_)_4_ was added. The solution was stirred at 120 °C for 32 h under a nitrogen atmosphere. After this, 0.04 mmol of 2-tributyl-stannylthiophene was added as chain closure, maintaining the reaction for 2 h at 120 °C. Then, 0.08 mmol of bromobenzene was added as a second chain closure, and the reaction was maintained for another 2 h at 120 °C. After this period, the temperature was lowered to 60 °C and 6 mL of toluene were added, leaving it under stirring for 1 h. Then the reaction was cooled to room temperature, and the polymer was precipitated in 120 mL of ice-cold methanol. The polymer was collected by filtration and subjected to Soxhlet extraction with methanol, acetone, hexane, and chloroform at the end. The polymer was recovered as a solid from the chloroform fraction by precipitation in ice-cold methanol. The synthetic route is shown in Figure 2. The resulting solid was dried under vacuum, yielding a product with a 90% recovery. Gel permeation chromatography (GPC) analysis revealed a weight-average molecular weight (*M*_w_) of 66.33 kDa, a number-average molecular weight (*M*_n_) of 32.32 kDa, and a polydispersity index (PDI) of 2.05.

**Figure 2:**
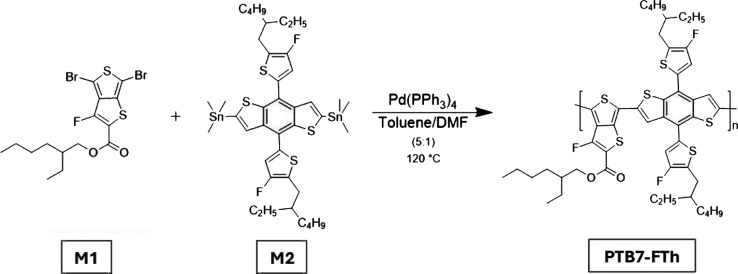
Synthetic route of PTB7-FTh.

### 2.3 Measurements

Ultraviolet–visible (UV–Vis) absorption spectra were recorded using a Shimadzu Corp (Japan) UV-2600i spectrophotometer with films of the samples deposited on glass substrates. The optical bandgap (Eg) was calculated using the wavelength at the onset of the highest absorbance (λ_onset_) and the Planck’s equation (*E* = *hc* λ^–1^), where *E* is energy (J), h is the Planck constant (*h* = 6.626 × 10^–34^ J s), *c* is the speed of light (2.998 × 10^8^ m s^–1^), and λ is the wavelength (nm). The electrochemical CV was conducted by a Metrohm Autolab (The Netherlands), where including ITO (used for working electrode), Ag/Ag^+^ electrode (reference electrode), and Pt wire (counter electrode) with a scanning rate of 50 mV s^–1^ in a 0.1 M Bu_4_NPF_6_ acetonitrile solution. The molecular weight of the polymer was performed on Agilent1260 Infinity II High-Temperature GPC equipment with a refractive index detector, using an Agilent (USA) PLgel 10 μm Mixed-B column (300 × 7.5 mm, PN: PL1110-6100), calibrated with narrow distributed polystyrene standards and eluted in trichlorobenzene at 160 °C. AFM measurements were performed to investigate the surface morphology of the active layers using a JPK Nanowizard Atomic Force Microscope (Bruker Nano GmbH, Berlin, Germany) operated in tapping mode. Although techniques such as GIWAXS and mobility measurements are well-established tools for deeper understanding of morphology and charge transport, they were not accessible in the present work due to our focus on scalable processes under ambient conditions. The current–voltage (*J*–*V*) characteristics of the devices were measured using a Keithley 2400 SourceMeter unit equipped with a multiplexer board (Tektronix Inc., USA), controlled by a computer and connected to a solar simulator with a xenon lamp (GreatSolar, Italy; AM 1.5G spectral match, AAA rating). The measurements were made under an irradiance of 1000 W m^–2^, calibrated with a silicon reference cell. The voltage scan was performed at 100 points between −1 and 1 V, after the samples were subjected to 1 min of light irradiation, in the equipment itself. All measurements were made at room temperature (<23 °C) and the samples were positioned in the sample holder in an N_2_ atmosphere and under a quartz glass. External quantum efficiency (EQE) measurements were carried out using a PTS-2-QE Quantum Efficiency/IPCE system (Sciencetech Inc., Canada). The system was calibrated with a certified reference silicon photodiode prior to measurements, and the spectra were recorded in the wavelength range of 280–1000 nm.

### 2.4 Device Fabrication

The behavior of the PTB7-FTh material was evaluated in different inverted-structure photovoltaic device systems, consisting of IMI/ZnO or SnO_2_/Active Layer/Modified PEDOT/Al. IMI refers to a transparent electrode based on a multilayer structure of metals and conductive oxides, such as Indium–Tin Oxide (ITO)/Metal (M)/ITO or Indium–Zinc Oxide (IZO)/Metal (M)/IZO, which provides improved electrical conductivity, optical transparency, and mechanical flexibility compared to conventional ITO electrodes. The structural specifications of each system are presented in Figure 3a. The devices were fabricated on flexible substrates PET-based already coated with a transparent conductive oxide, as mentioned previously. On these substrates, the electron transport layers, active layer, and hole transport layers were sequentially deposited using the blade coating method. To complete the devices, a silver layer was evaporated, serving as the top electrode.

**Figure 3:**
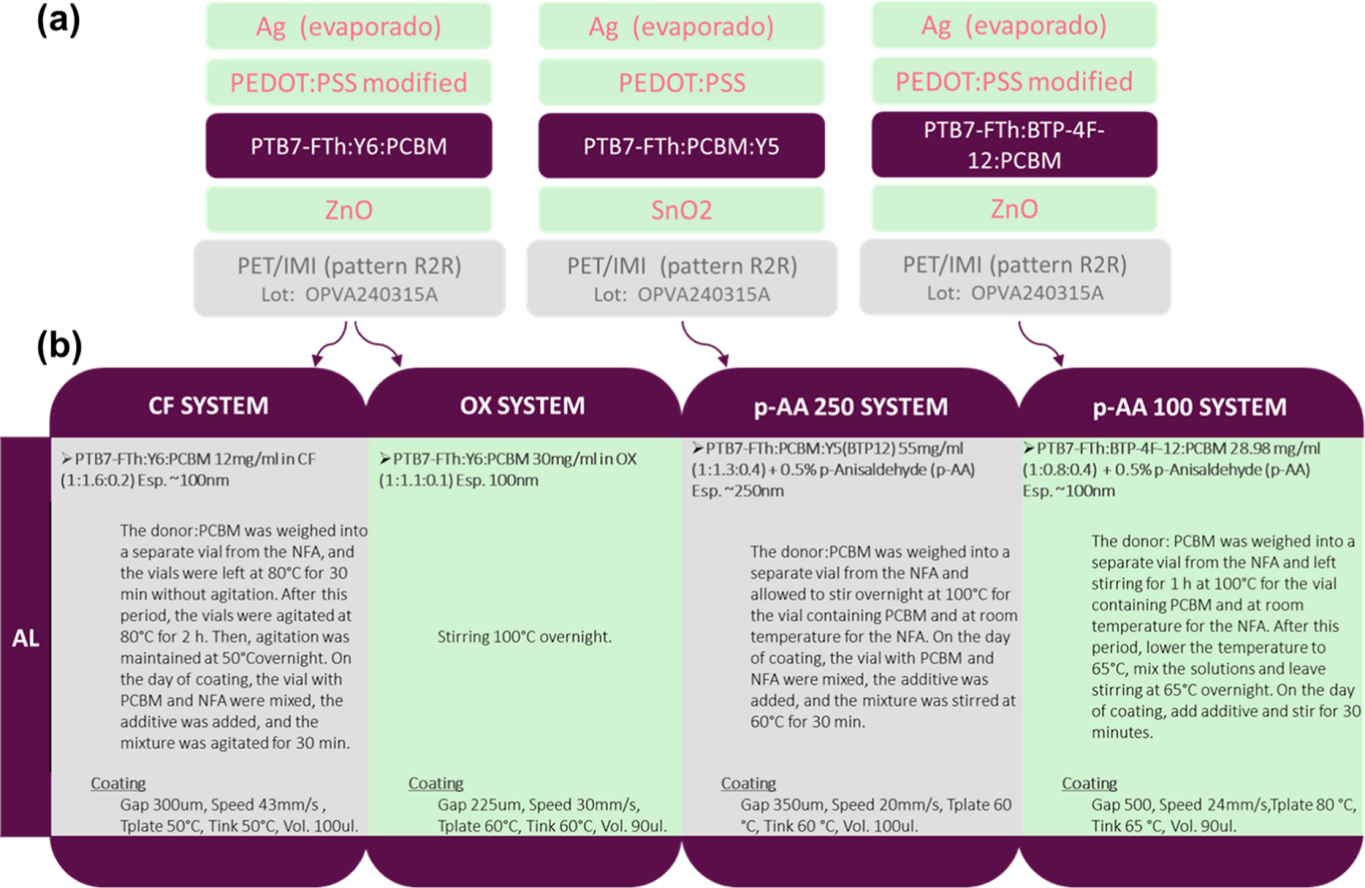
(a) Architecture of the evaluated systems; (b) deposition parameters of the active layers used in the evaluated systems.

The ZnO films were processed on the blade coating plate at 45 °C using 150 μL of zinc oxide (ZnO) solution supplied by Infinity PV. The solution was prepared as a sol–gel diluted in isopropanol, with an initial concentration of 5.6 wt %, which was adjusted to 2.1% after dilution. The solution was continuously stirred at room temperature throughout the process. For deposition, the blade gap was set to achieve a 700 μm, and the deposition speed was 5 mm min^–1^. Immediately after deposition, the films underwent thermal annealing in air using a hot plate set to 120 °C for 3 min. The active layer was then deposited onto the ZnO according to the specific details of each system, as shown in Figure 3b.

The last layer deposited using the blade coating technique was the hole transport layer (HTL). The modified PEDOT films were processed on the blade coating plate at 65 °C using 150 μL of the HTL-X solution supplied by Raynergy Tek Inc. This material was specifically designed for nonfullerene acceptor-based devices. The original solution was diluted in isopropanol at a 1:4 ratio (solvent/solution) and continuously stirred at room temperature throughout the process. The blade gap was set to maintain a 500 μm, and the deposition speed was 4 mm min^–1^.

After deposition, the contacts were cleaned with xylene to remove the layers at the contact points of the cells, ensuring proper connection between the silver electrode and the IMI. Subsequently, a 200 nm-thick silver layer was deposited by thermal evaporation under a pressure below 3 × 10^–6^ mbar. The evaporation rate was set to 1 Å s^–1^ for the first 10 nm and 2 Å s^–1^ for the remaining 190 nm. The active area of 8 cells for single device was 0.55 cm^2^ each.

## 3 Results and Discussion

### 3.1 Polymer Synthesis and Characterization

The polymer PTB7-FTh was synthesized via a Stille coupling reaction. In addition to exhibiting good solubility in chloroform, the resulting material also demonstrated excellent solubility in o-xylene, which is crucial for large-scale production. The number-average molecular weight (*M*_n_), weight-average molecular weight (*M*_w_), and polydispersity index (PDI) of PTB7-FTh were found to be 32.32 kDa, 66.33 kDa, and 2.05, respectively, as measured by gel-permeation chromatography (GPC) eluted in trichlorobenzene at 160 °C using a column calibrated with narrow-distribution polystyrene standards.

Figure 4a shows the UV–vis absorption spectrum of PTB7-FTh in solid film form. The polymer exhibits strong absorption in the 300–400 nm and 450–800 nm regions, which can be attributed to π–π transitions of the polymer backbone and intramolecular charge-transfer (ICT) processes, respectively. A prominent absorption peak is observed at 675 nm, indicating efficient light harvesting in the visible to near-infrared region. The absorption profile of PTB7-FTh is similar to that of its structural analogue PTB7-Th,^15^ with a slight red shift in the main absorption band. This shift suggests that modifications in the polymer structure, particularly in the acceptor unit, enhance molecular interactions and extend conjugation, which could influence charge separation and transport properties in photovoltaic devices. The optical bandgap (Eg^opt^) of PTB7-FTh was estimated to be 1.55 eV, which is slightly lower than that of PTB7-Th (1.57 eV),^16,17^ and also lower than that of PBFTT (1.64 eV), a structurally related polymer synthesized by Chang and colleagues in 2018. The reduction in bandgap suggests improved photon absorption in the lower-energy region, potentially leading to enhanced photocurrent generation in OPV devices. Furthermore, the absorption onset and spectral broadening of PTB7-FTh highlight its potential for effective pairing with nonfullerene acceptors (NFAs), optimizing exciton generation and dissociation.

**Figure 4:**
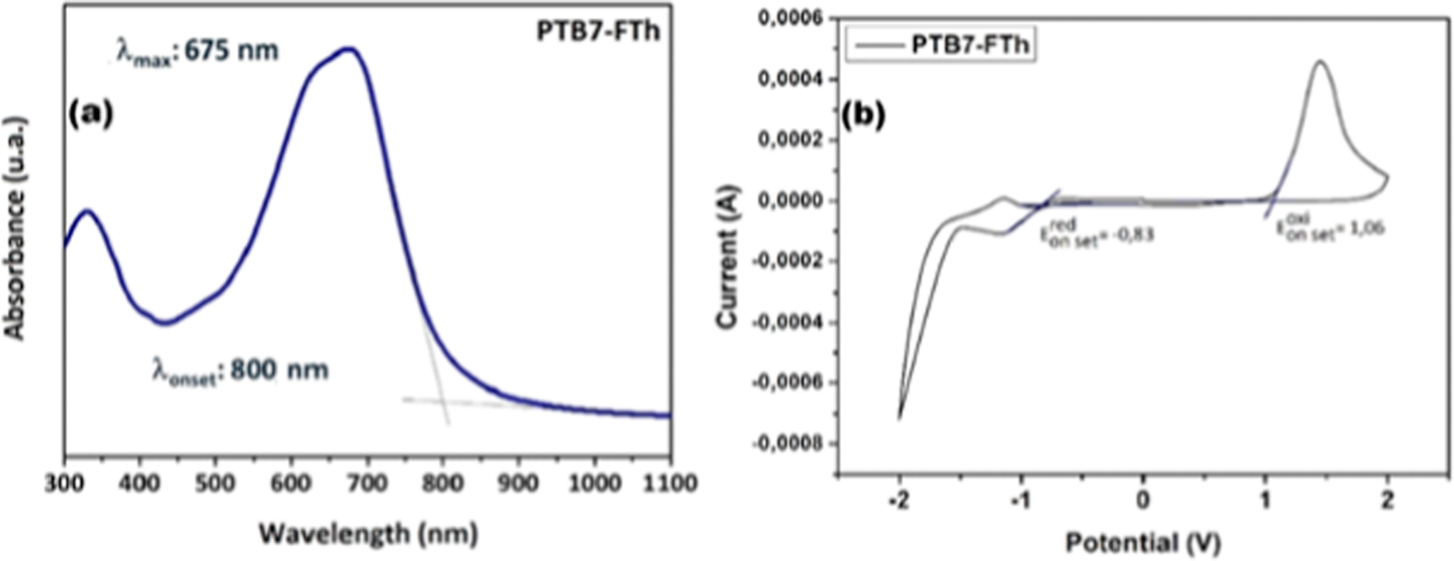
(a) UV–Vis absorption spectrum of PTB7-FTh thin film; (b) electrochemical response of PTB7-FTh used for energy level determination.

Figure 4b presents the electrochemical cyclic voltammetry (CV) data, which was used to determine the HOMO and LUMO energy levels of PTB7-FTh. The onset oxidation potential (ϕox) of PTB7-FTh is 1.06 V vs. Ag/Ag^+^, while the onset reduction potential (ϕred) is −0.83 V vs. Ag/Ag^+^. Using the standard eqs [70], HOMO = −*e*(ϕox +4.4) (eV) and LUMO = −*e*(ϕred +4.4) (eV), the HOMO and LUMO energy levels of PTB7-FTh were determined to be −5.46 eV and −3.57 eV, respectively, For comparison, PTB7-Th exhibits HOMO and LUMO levels of −5.22 eV and −3.64 eV, respectively.^15^ The deeper HOMO energy level of PTB7-FTh, which is 0.24 eV lower than that of PTB7-Th, can be attributed to the electron-withdrawing effect of the fluorine substituents. Fluorination enhances molecular dipole interactions and stabilizes the electronic structure, leading to a deeper HOMO level. The lower HOMO level (−5.47 eV) observed by Chang and collaborators (2018) was attributed to the introduction of fluorine atoms into the polymer chain, which enhanced the electron-withdrawing character of the material. This modification is particularly advantageous for achieving higher *V*_oc_ in OPVs, as a deeper HOMO level of the donor material increases the energy difference between the HOMO of the donor and the LUMO of the acceptor, thereby improving the driving force for charge separation. Du and collaborators (2018) also obtained a material with a deeper HOMO (−5.30 eV) by synthesizing a similar material. However, in addition to fluorine (F), the authors also introduced an alkylthiolation branching, which could contribute negatively to the HOMO level not being as deep as in materials with only alkoxy branching, since the presence of the sulfur atom in the chain could interfere with the electronic distribution of the molecule. Although sulfur is electronically donating, it may have a more complex interaction with other substituent groups, influencing the stability of the molecular orbitals and resulting in a less negative HOMO level.^14^

The observed energy level tuning via fluorination highlights the potential of PTB7-FTh as a promising donor material for nonfullerene organic solar cells, where precise control of energy levels is essential for optimizing device performance.

### 3.2 Fabrication of OPV Devices

To investigate the photovoltaic properties of PTB7-FTh in different systems and determine the optimal components for device fabrication, four distinct architectures were analyzed. The layers used in device construction are shown in Figure 3a. For the CF and OX systems (described in Figure 3b), the same layer structure was employed; however, the active layer compositions differed, with PTB7-FTh:Y6:PC_70_BM mass ratios of 1:1.6:0.2 for the CF system and 1:1.1:0.1 for the OX system. Additionally, different solvents were used: chloroform for the CF system and o-xylene for the OX system. The use of o-xylene as a processing solvent represents a significant step toward achieving compatibility with large-scale production techniques.^18,19^ The variations in device configurations and fabrication parameters are systematically summarized in Figure 3b, allowing a comprehensive comparison of the approaches investigated. O-xylene was also employed as the processing solvent in two additional systems, which incorporated further modifications, such as the use of *p*-anisaldehyde (*p*-AA) as an additive, alternative acceptor materials, and variations in active layer thickness. As an additive, *p*-anisaldehyde plays a crucial role in optimizing the active layer morphology by promoting favorable phase separation, enhancing molecular ordering, and facilitating charge transport.^20^ It suppresses excessive aggregation, increases charge carrier mobility, and reduces recombination losses. Moreover, *p*-AA can modulate energy levels and improve film uniformity, which is particularly advantageous for scalable deposition techniques such as blade coating. Collectively, these effects contribute to higher efficiency and improved operational stability in OPV devices.

The selection of o-xylene as a processing solvent is particularly relevant, as it not only facilitates compatibility with large-scale production techniques but also offers a safer and more environmentally sustainable alternative to conventional chlorinated solvents. These optimizations aim to enhance device performance while ensuring industrial feasibility.^21^ Considering the importance of studying alternative solvents for the development of organic devices, Kumari et al. (2020) achieved efficiencies of 11.02% and 11.76% in bulk heterojunction OSCs with a donor:acceptor blend containing PTB7-Th and fullerene and nonfullerene acceptors, respectively. These results were obtained in devices with an area of 0.13 cm^2^, using a halogen-free processing system with o-xylene/*N*-methylpyrrolidone.^22^ Beyond their environmental significance and potential for the production of efficient devices, Mazzolini et al. (2024) demonstrated that devices processed with o-xylene exhibit a 40% reduction in the bimolecular recombination coefficient compared to those processed with CB, along with a 70% increase in effective mobility.^23^

The systematic comparison of device configurations and fabrication parameters, presented in Figure 3b, highlights the impact of these modifications, providing valuable insights into the role of solvent choice, material composition, and processing conditions in optimizing OPV device efficiency and scalability. Most OPV devices reported in the literature using PTB7-Th employ only PC_71_BM as the acceptor material. Their fabrication is typically carried out entirely in an inert atmosphere using the spin-coating technique, achieving an average power conversion efficiency of approximately 10%.^11,24,25^

In this study, the investigated polymer has a similar backbone to PTB7-Th but incorporates two fluorine atoms. As expected, fluorination lowered the HOMO energy level, directly influencing the *V*_oc_ of the fabricated devices. In addition to this structural modification, which was designed to enhance photovoltaic performance, this work also aims to bridge the gap between laboratory-scale device fabrication and industrial production. To achieve this, this work employed blade coating in an ambient atmosphere for film deposition, a method that more closely resembles scalable roll-to-roll slot-die manufacturing. Furthermore, the fabricated devices were 0.15 cm^2^ larger than those typically evaluated in previous studies, further aligning with industrially relevant processing conditions.

The devices were fabricated with an inverted device structure, as shown in Figure 3a. First, the influence of solvents, donor/acceptor/acceptor (D/A/A) mass ratios, thickness, and thermal treatment on the active layer was investigated in relation to device performance. Figure 5a presents the current density–voltage (*J*–*V*) characteristics of the best OPV curves under AM 1.5G illumination at 100 mW cm^–2^, with the corresponding photovoltaic parameters summarized in Table 1. Additionally, the maximum values obtained for each parameter (*V*_oc_, *J*_sc_, FF, and PCE), both before and after thermal treatment, are summarized in Table 2 and illustrated in the box plot of Figure 6, which shows the distribution of photovoltaic parameters across multiple cells from the fabricated devices. The optimal results were achieved after annealing, using the ternary blend consisting of the donor polymer and two acceptors, Y6 and PC_71_BM, solubilized in chloroform, with a donor-to-acceptor ratio (D/A/A) of 1:1.6:0.2. Under these conditions, the devices exhibited a power conversion efficiency (PCE) of 5.03%, with a *V*_oc_ of 0.76 V, a *J*_sc_ of 13.39 mA cm^–2^, and an FF of 49.22%. These results are comparable to those of PTB7-Th:ITIC-based devices fabricated entirely in an inert atmosphere (*V*_oc_ of 0.81 V, *J*_sc_ of 14.2 mA cm^–2^, FF of 59.1%, and a PCE of 6.8%).^26–28^ They demonstrate the viability of the ternary blend approach, where the combination of a high-performance nonfullerene acceptor (Y6) with the fullerene derivative (PC_71_BM) contributes to improved charge transport and morphology optimization. The observed performance is particularly notable given that these devices were processed under noninert conditions, which typically lead to increased defect densities and reduced charge transport efficiency.

**Figure 5:**
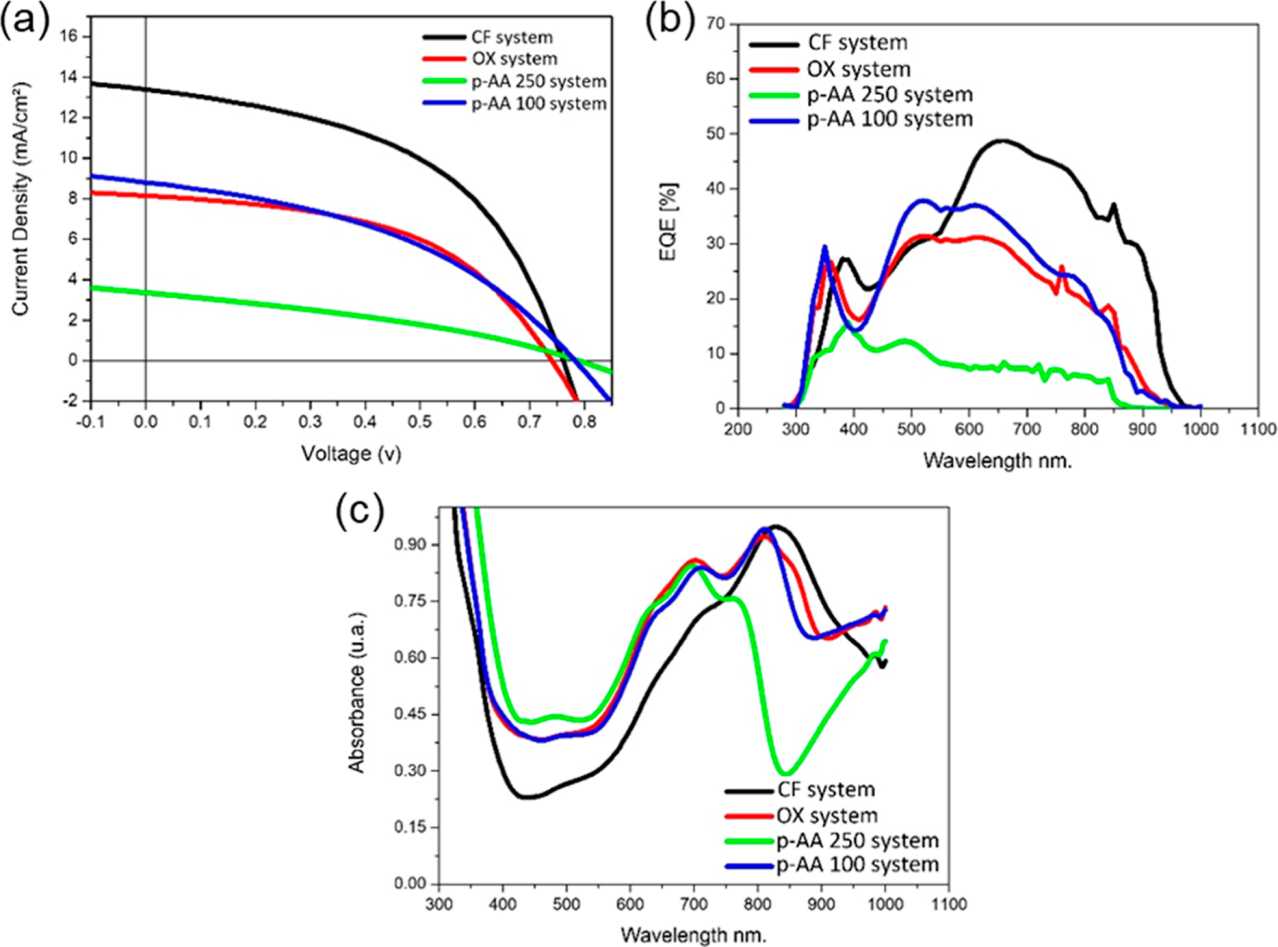
(a) Best *J*–*V* curves of the PSCs based on PTB7-FTh with different systems; (b) EQE curve of the corresponding device; (c) absorption spectra of the corresponding active layer.

**Table 1:** Photovoltaic Parameters of the Best OPV Cells

Best OPV Cells	*V*_oc_ [V]	*J*_sc_ [mA cm^–2^]	FF [%]	PCE [%]
CF system	0.76	13.39	49.22	5.03
OX system	0.74	8.15	49.55	3.00
*p*-AA 250 system	0.79	3.36	33.82	0.90
*p*-AA 100 system’	0.78	8.81	41.18	2.85

**Table 2:** Maximum Values of the Photovoltaic Parameters Analyzed in Each System before and after Annealing

system	*V*_oc_ (V)	*J*_sc_ (mA cm^–2^)	FF (%)	PCE (%)
as-coated				
CF	0.75 (0.67 ± 0.08)	14.10 (12.61 ± 0.77)	43.80 (37.54 ± 3.73)	4.56 (3.23 ± 0.81)
OX	0.73 (0.67 ± 0.06)	8.00 (7.39 ± 0.32)<	43.90 (37.91 ± 3.23)	2.42 (1.90 ± 0.34)
*p*-AA 250	0.80 (0.77 ± 0.03)	3.50 (3.04 ± 0.39)	34.80 (33.11 ± 0.54)	0.90 (0.78 ± 0.11)
*p*-AA 100	0.79 (0.78 ± 0.01)	8.90 (8.44 ± 0.34)	41.40 (39.40 ± 1.11)	2.85 (2.59 ± 0.13)
postannealed in GB - 80 °C for 3 min				
CF	0.764 (0.725 ± 0.050)	14.10 (13.07 ± 0.56)	49.20 (42.96 ± 3.93)	5.03 (4.11 ± 0.73)
OX	0.745 (0.716 ± 0.027)	8.30 (7.75 ± 0.30)	49.50 (43.71 ± 3.14)	3.00 (2.43 ± 0.27)
*p*-AA 250	0.811 (0.760 ± 0.055)	3.20 (2.73 ± 0.42)	34.50 (33.24 ± 1.34)	0.84 (0.69 ± 0.13)
*p*-AA 100	0.786 (0.781 ± 0.003)	9.00 (8.43 ± 0.35)	41.80 (39.64 ± 1.23)	2.82 (2.61 ± 0.14)

**Figure 6:**
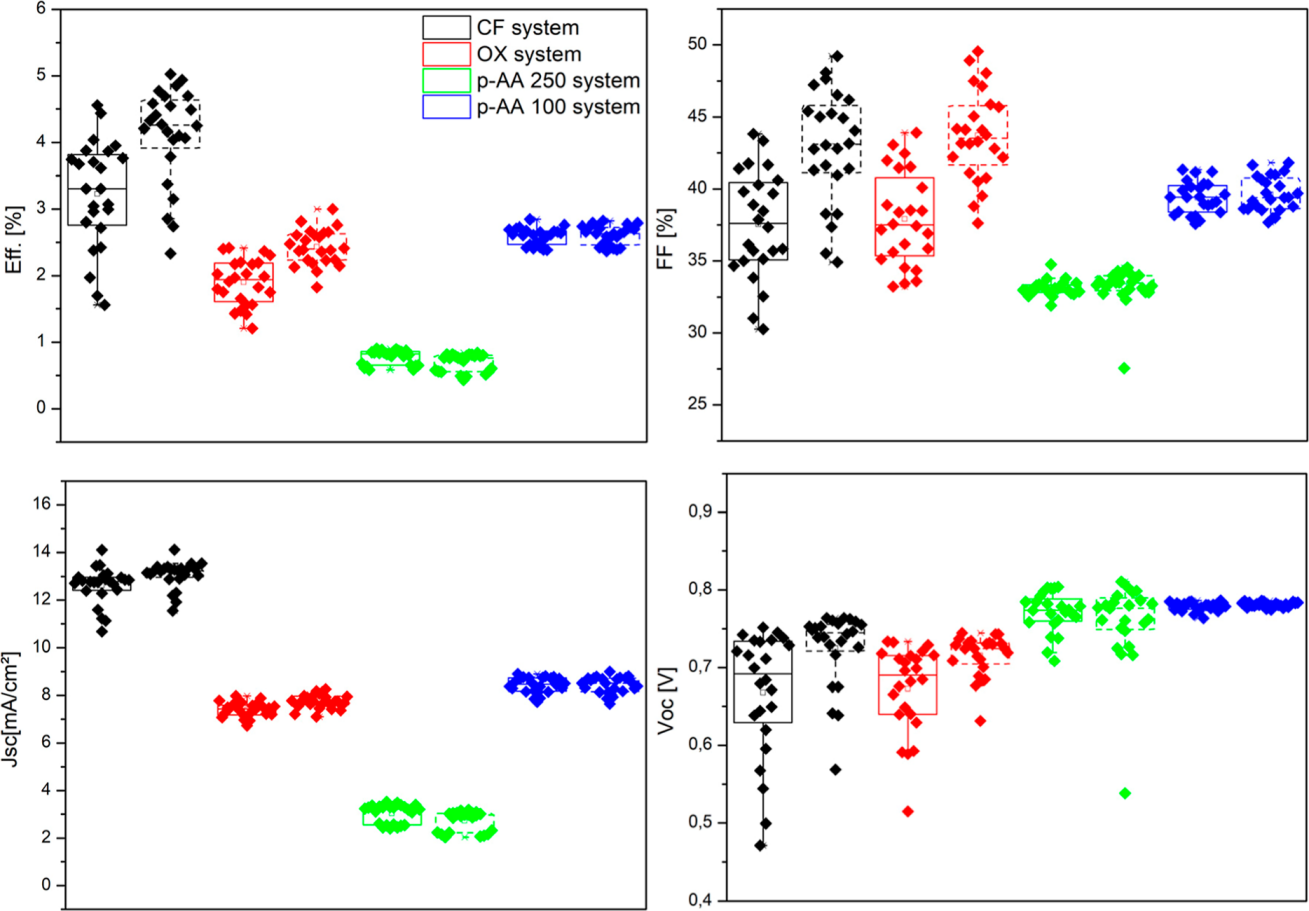
Box plot showing the distribution of photovoltaic parameters (*V*_oc_, *J*_sc_, FF, and PCE) across multiple cells from the fabricated devices, before and after thermal treatment.

Although the obtained power conversion efficiency (PCE) of 5.03% is lower than the 6.8% reported for PTB7-Th:ITIC-based devices fabricated entirely under inert atmosphere (*V*_oc_ = 0.810 V, *J*_sc_ = 14.20 mA cm^–2^, FF = 59.10%),^26^ the performance of the PTB7-FTh-based devices remains promising when considering the more realistic and industrially relevant processing conditions used in this study. Devices were fabricated under in larger active area, diverging from standard lab-scale methods. These factors typically lead to lower efficiencies but better reflect the challenges of industrial implementation. Therefore, this work provides valuable insights into the translation of conjugated polymer design into practical device architectures, bridging the gap between laboratory research and scalable organic photovoltaic manufacturing—a key hurdle for commercialization.

The slightly lower *V*_oc_ (0.76 V vs. 0.81 V) may be attributed to the deeper HOMO level of PTB7-FTh, induced by fluorination, which affects the energy alignment with the acceptor materials. Furthermore, the reduced FF (49.22% vs. 59.10%) suggests that interfacial recombination and charge transport limitations could be further optimized by fine-tuning the blend composition, solvent additives, and postprocessing conditions. Despite these challenges, the comparable *J*_sc_ (13.39 mA cm^–2^ vs. 14.20 mA cm^–2^) suggests that the absorption properties and exciton generation capabilities of PTB7-FTh remain effective under the tested conditions. These findings highlight the potential of ternary blend systems and noninert processing methods for advancing scalable OPV fabrication while maintaining competitive efficiency levels.

The superior *V*_oc_ was observed in devices with the highest PC_71_BM concentration, specifically in *p*-AA 250 (0.81 V) and *p*-AA 100 (0.79 V). In contrast, the lowest *V*_oc_ was observed in the system with the lowest PC_71_BM concentration (OX system), suggesting that the energy level alignment provided by the fullerene acceptor plays a more decisive role in facilitating charge separation and transport compared to the nonfullerene acceptors (NFAs) employed. The superior alignment of PC_71_BM energy levels likely contributes to improved charge separation and reduced recombination losses, ultimately leading to higher open-circuit voltages.

Among the two systems with the highest performance in this parameter, *p*-AA 250 stood out, despite exhibiting the lowest overall efficiency among the studied devices. This lower efficiency is likely attributed to the increased thickness of the active layer, which can hinder charge transport and promote recombination. For the polymer evaluated, PC_71_BM combined with Y5 appears to be the most suitable acceptor system, although, based on the energy levels presented in Figure 7, this choice is not the most intuitive due to the small difference between the LUMO levels of PTB7-FTh and Y5. Although *p*-AA 250 achieved the highest *V*_oc_, it exhibited the poorest performance in terms of current generation, with a *J*_sc_ of only 3.50 mA cm^–2^. This significant reduction in *J*_sc_ is likely due to the increased active layer thickness (250 nm) compared to the other systems (∼100 nm), which may have negatively impacted exciton dissociation and charge transport, thereby increasing recombination losses.

**Figure 7:**
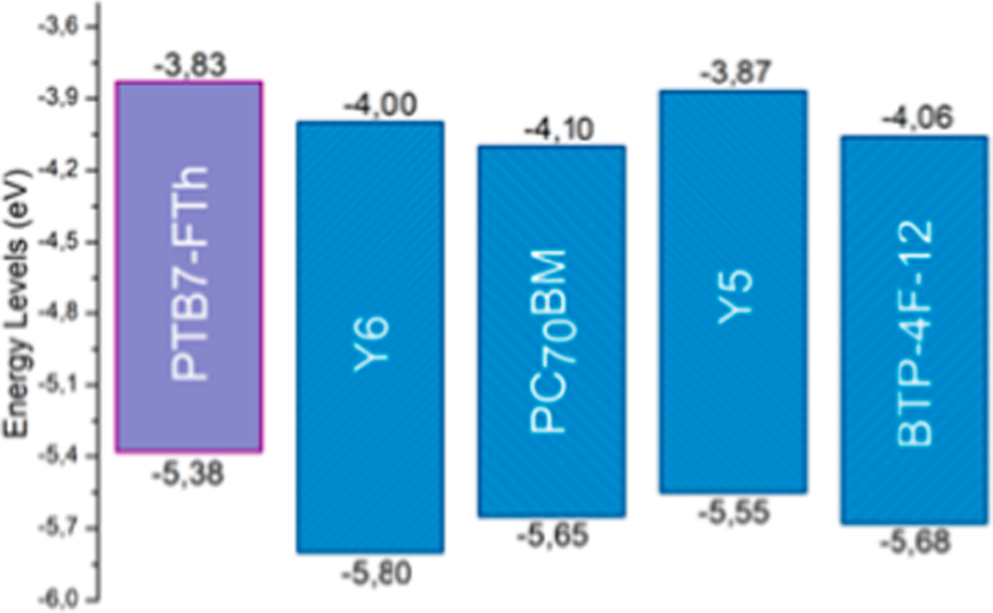
Energy levels of the donor polymer and the acceptor materials used in the construction of the active layers.

Additionally, the narrow absorption range of the active layer components (Figure 5c) may have further contributed to the lower current generation. In contrast, the CF system exhibited the highest *J*_sc_ (14.10 mA cm^–2^), indicating superior charge generation and extraction efficiency. This system was the only one to display a red-shift in absorption, correlating with enhanced light-harvesting capability and broader spectral coverage. While both CF and *p*-AA 250 systems had higher acceptor concentrations, the excessive thickness of the *p*-AA 250 active layer likely hindered efficient photon absorption and charge transport, leading to reduced exciton dissociation and increased recombination losses. Conversely, the CF system combined favorable donor–acceptor energy alignment with an optimized film morphology, enabling more efficient charge transport.

The OX and *p*-AA 100 systems showed similar *J*_sc_ values (8.30 and 9.00 mA cm^–2^, respectively), consistent with their absorption profiles (Figure 5c). Regarding the fill factor (FF), Table 2 shows that both the CF and OX systems benefited significantly from thermal annealing, with FF increasing from ∼44% to ∼49%. This improvement is attributed to the morphology optimization of Y6 upon annealing, which enhances charge mobility and reduces recombination.

Notably, variations in *J*_sc_ across systems were closely linked to morphological differences observed by AFM (Figure 8) and absorption characteristics. The CF film, which exhibited smoother morphology and finer phase separation, supported more effective exciton dissociation and charge collection. In contrast, the *p*-AA 250 system, despite its higher PCBM content, displayed lower *J*_sc_, likely due to suboptimal morphology and excessive thickness. EQE measurements further supported this trend, with devices showing broader and red-shifted spectral response also presenting higher photocurrents.

**Figure 8:**
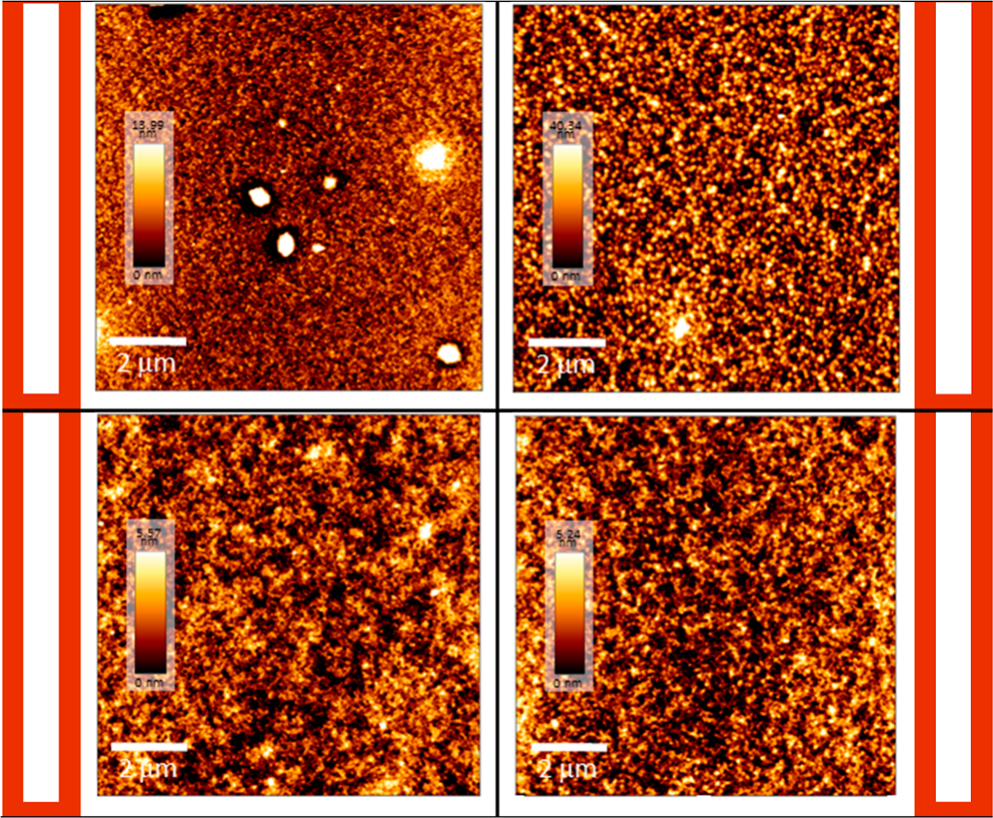
AFM topography images of the active layer films of the different systems evaluated.

Among all systems, *p*-AA 100 exhibited the most stable FF, unaffected by thermal annealing, suggesting that its film morphology was already optimized during deposition. In contrast, *p*-AA 250 displayed the lowest FF and showed no improvement upon annealing, reinforcing the detrimental effect of increased thickness on charge transport. Overall, the CF system demonstrated the best performance among the evaluated configurations, highlighting the importance of optimized donor–acceptor ratios, active layer morphology, and processing conditions in maximizing OPV efficiency. The EQE response in the wavelength range from 300 to 1000 nm aligns well with the PCE trends observed in the devices. The highest EQE value, reaching 49%, was recorded for the CF system, indicating efficient photon-to-electron conversion across the absorption spectrum. In contrast, the lowest EQE value of around 16% was observed for the *p*-AA 250 system, suggesting lower charge generation and extraction efficiency. The variations in EQE among different systems can be attributed to differences in active layer morphology, charge transport properties, and donor–acceptor energy level alignment. Additionally, the spectral response confirms the effectiveness of specific donor–acceptor combinations in enhancing photocurrent generation within the active layer.

To analyze the phase separation differences of the active layer films in different systems and study the impact of surface morphologies on photovoltaic parameters, the films were characterized by atomic force microscopy (AFM). The topographic images of the active layer films, presented in Figure 8, show that the film processed with chloroform as the solvent (CF System) and chloronaphthalene (CN) as the additive exhibited the most distinct morphology among the four systems. This film presents submicrometric heterogeneities that suggest an organization of the acceptor material. The surface presents mostly a continuous portion with fine-sized topographic elements. The topography of the region analyzed varies from 0 to 13.99 nm, suggesting low roughness, which is favorable for the transport of electrical charges. Gavim et al. (2022) investigated the effect of chloronaphthalene (CN) on the morphology of PTB7-Th:PDIC5 films, observing that the addition of 0.5% CN resulted in a more homogeneous surface with reduced roughness. The greatest impact of CN was on the distribution of PDIC5 along the active layer, which led to a reduction in phase segregation and an improvement in the performance of the acceptor material, as evidenced by the decrease in root-mean-square roughness (Rrms) from 13 to 7 nm.^29^

On the other hand, in the image of the OX system using o-xylene as the solvent without any additive, the topography of the analyzed region varies from 0 to 40.34 nm, suggesting an increase in roughness for this system. Despite not presenting large heterogeneities, the topographic elements in this continuous film have a rounded shape and are more evident than those in the continuous region of the CF. This morphological change negatively impacts *J*_sc_, as it may generate charge traps and hinder the effective diffusion of excitons, leading to a loss of efficiency. Liu et al. (2023) investigated the effect of additives on the morphology of blend films, noting that the film without additives was more rugged compared to those processed with additives. The films treated with two specific additives exhibited a smoother surface, which facilitated the formation of a better ohmic contact, resulting in a higher *J*_sc_.^30^

In the systems with o-xylene and the addition of *p*-anisaldehyde as an additive (*p*-AA 250 and *p*-AA 100 systems), an improvement in roughness was observed, also keeping the surfaces more homogeneous. However, for the *p*-AA 250 system, with a film thickness of 250 nm, charge dissociation became inefficient, leading to a significant loss in current. In contrast, for the *p*-AA 100 system, despite the improved morphology, the performance was similar to that of the OX system, which did not use an additive, with both systems showing comparable efficiency.

Despite the higher roughness of the OX system, the choice of Y6 as the main acceptor provided a broader absorption, as observed in the absorption spectrum of the active layer (Figure 5c). The enhanced light absorption was sufficient to offset the high surface roughness, enabling the OX system to reach an efficiency comparable to that of the *p*-AA 100 system, despite the latter exhibiting a more favorable morphology.

## 4 Conclusions

In this study, a D–A copolymer named PTB7-FTh was successfully synthesized and applied as a donor material in OPV devices fabricated via blade coating under an ambient atmosphere. The results demonstrated that the use of a ternary blend with Y6 and PC_71_BM as acceptors in a 1:1.6:0.2 mass ratio led to the highest PCE of 5.03%, highlighting the effectiveness of mixed acceptor systems in improving charge transport and light absorption. The selection of o-xylene as a processing solvent proved advantageous, offering better compatibility with scalable manufacturing processes while maintaining competitive efficiency levels. Furthermore, the influence of active layer thickness was evident, as devices with an optimized thickness of approximately 100 nm exhibited superior performance, whereas thicker films (such as in the *p*-AA 250 system, even with *p*-anisaldehyde as additive) faced charge transport limitations and increased recombination losses.

Thermal annealing at 80 °C for 3 min further enhanced device performance, particularly in systems utilizing Y6, which showed improvements in FF and overall efficiency. However, some systems, such as *p*-AA 100, exhibited stable performance even without annealing, indicating that morphology control during deposition played a crucial role. The fluorination in PTB7-FTh led to a deeper HOMO energy level compared to PTB7-Th, contributing to an increase in *V*_oc_ and suggesting that strategic molecular modifications can further optimize energy level alignment for improved device performance. Overall, this study demonstrates the viability of PTB7-FTh as a donor polymer in scalable OPV fabrication, bridging the gap between laboratory-scale research and industrial production. The findings highlight the importance of solvent selection, acceptor composition, and processing conditions in optimizing OPV efficiency. Future work should focus on further refining processing parameters and exploring alternative nonfullerene acceptors to enhance charge carrier dynamics and achieve efficiencies beyond 5%, reinforcing the potential of blade-coated OPVs for commercial applications. Additionally, incorporating advanced morphological and structural characterization techniques, such as GIWAXS and TEM, will be crucial for deepening the understanding of phase separation, crystallinity, and their impact on device performance.
